# Correction to “Switchable Behavior of Ru–TiO_2_ Catalysts in HMF Conversion”

**DOI:** 10.1021/acssuschemeng.5c11373

**Published:** 2025-11-07

**Authors:** Babar Amin, Jaroslav Aubrecht, Oleg Kikhtyanin, Evgeniya Grechman, Gustavo Andrade Silva Alves, Alberto Tampieri, Karin Föttinger, Marcin Jędrzejczyk, Agnieszka M. Ruppert, Francisco Ruiz-Zepeda, David Kubička

The following sentences are being added to the Acknowledgements: This research was funded in part by the Austrian Science Fund (FWF, 10.55776/F8100). For open access purposes, the author has applied a CC BY public copyright license to any author-accepted manuscript version arising from this submission.

